# Resolvin D1 Modulates the Intracellular VEGF-Related miRNAs of Retinal Photoreceptors Challenged With High Glucose

**DOI:** 10.3389/fphar.2020.00235

**Published:** 2020-03-06

**Authors:** Rosa Maisto, Maria Consiglia Trotta, Francesco Petrillo, Sara Izzo, Giovanna Cuomo, Roberto Alfano, Anca Hermenean, Jorge Miquel Barcia, Marilena Galdiero, Chiara Bianca Maria Platania, Claudio Bucolo, Michele D’Amico

**Affiliations:** ^1^Section of Pharmacology, Department of Experimental Medicine, University of Campania “Luigi Vanvitelli”, Naples, Italy; ^2^Department of Experimental Medicine, University of Campania “Luigi Vanvitelli”, Naples, Italy; ^3^Multidisciplinary Department of Surgical and Dental Specialties, University of Campania “Luigi Vanvitelli”, Naples, Italy; ^4^Department of Precision Medicine, University of Campania “Luigi Vanvitelli”, Naples, Italy; ^5^Department of Advanced Medical and Surgical Sciences, University of Campania “Luigi Vanvitelli”, Naples, Italy; ^6^Institute of Life Sciences, Vasile Goldis Western University of Arad, Arad, Romania; ^7^School of Medicine, Catholic University of Valencia “Saint Vicente Martir”, Valencia, Spain; ^8^Department of Biomedical and Biotechnological Sciences, School of Medicine, University of Catania, Catania, Italy

**Keywords:** retinal photoreceptors, exosomes, miRNAs, resolvin D1, VEGF

## Abstract

Stimulation of retinal photoreceptors with elevated glucose concentration (30 mM) for 96 h, served as diabetic retinopathy *in vitro* model to study Resolvin D1 (50 nM) effects on neovascularization. VEGF and anti-angiogenic miR-20a-3p, miR-20a-5p, miR-106a-5p, and miR-20b expression was assessed either in photoreceptors exposed to HG or in exosomes released by those cells. High glucose increased VEGF levels and concurrently decreased anti-angiogenic miRNAs content in photoreceptors and exosomes. RvD1 reverted the effects of glucose damage in photoreceptors and exosomal pro-angiogenic potential, tested with the HUVEC angiogenesis assay. By activating FPR2 receptor, RvD1 modulated both the expression of anti-angiogenic miRNA, which decrease VEGF, and the pro-angiogenic potential of exosomes released by primary retinal cells. HUVEC transfection with miR-20a-3p, miR-20a-5p, miR-106a-5p, and miR-20b antagomirs, followed by exposure to exosomes from photoreceptors, confirmed the VEGF-related miRNAs mechanism and the anti-angiogenic effects of RvD1.

## Introduction

Diabetic retinopathy (DR) after progression from non-proliferative stage is characterized by vitreo-retinal neovascularization. When abnormal this may erupte through the surface of the retina or into the vitreous gel and hemorrhage into the vitreus space resulting in macular edema and vision loss (Crawford et al., 2009; Cho et al., 2013). The risk of diabetic microvascular alterations, including DR, increases with poor glycemic control (Fullerton et al., 2014). Besides glycemia or glycated hemoglobin and albumin, other biomarkers, would be diagnostic and prognostic of DR (Domingueti et al., 2016). Our previous study showed that some microRNAs (miRNAs) such as miR-20a-3p, miR-20a-5p, miR-106a-5p, and miR-20b were significantly downregulated in mouse serum and retina after 5 and 10 weeks of diabetes (animal model of early DR). In this animal model, hyperglycemia with dysregulated miRNAs induced detrimental ultrastructural retinal modification, also in the neuronal retina. Dysregulation of these miRNAs correlated with increased Vascular Endothelial Growth Factor (VEGF) and decreased Brain-Derived Neurotrophic Factor (BDNF) levels in diabetic retinas, before appearance of retinal microaneurysms (Platania et al., 2019).

Resolvin (RvD1), a metabolite of ω3-polyunsaturated fatty acid (PUFA), is a pro-resolving lipid mediator, which was found to inhibit inflammation in DR models (Shi et al., 2016; Yin et al., 2017). Furthermore, ω3-PUFA and related metabolites (RvD1, RvE1, and neuroprotectinD1) protected the mice retina from neovascularization induced by ischemia in a model of retinopathy induced by oxygen (Connor et al., 2007). Interestingly, it was found that RvD1 is capable to modulate the expression of several miRNAs by activating the N-formyl peptide receptor 2 (FPR2, alias ALX) (Recchiuti and Serhan, 2012; Bisicchia et al., 2018). With these premises, we hypothesized that RvD1 could exert anti-angiogenic effects through activation of its receptor FPR2, by modulation of anti-angiogenic miRNAs (targeting VEGFA) and decrease of VEGFA levels. To test our hypothesis, an *in vitro* model of DR such as retinal photoreceptors stimulated with elevated glucose concentrations was used. Cells were treated with RvD1 and Boc2, a FPR2 antagonist. VEGF and anti-angiogenic miRNAs expression levels were assessed either in photoreceptors exposed to HG or in exosomes released by those cells. Here we found that RvD1, by modulating the expression of ROS-induced nuclear factor kappa-light-chain-enhancer of activated B cells (NF-kB) signaling causes changes of the intracellular miRNAs miR-20a-3p, miR-20a-5p, miR-106a-5p, and miR-20b of photoreceptors stimulated with high glucose, and the release of these miRNAs-containing exosomes.

## Materials and Methods

### Primary Retinal Cells

Retinal cells were isolated following the protocol described by Maisto et al. (2017) for retina collection.

A combination of ketamine (100 mg/kg) and medetomidine (0.25 mg/kg) was used to anesthetize three weeks old C57BL/6J mice (*n* = 10) (Harlan, Cat# JAX_000664, RRID: IMSR_JAX:000664), in order to perform the eye enucleation; then, animals were sacrificed by cervical dislocation. The Animal Ethics Committee of University of Campania “Luigi Vanvitelli” approved all the experimental procedures (Protocol Number 2108). Italian (D.L. 116/92) and European Commission (Directive 2010/63/EU) guidelines were followed for the animal care.

After eye enucleation, the retina was dissected under sterile conditions using the enzymes trypsin and collagenase A. After dissociation, primary retinal cells were collected by centrifugation and resuspended in Eagle’s minimum essential medium (MEM) supplemented with 26 mM NaHCO_3_, 25 mM HEPES, 10% heat-inactivated fetal bovine serum, penicillin (100 U/ml) and streptomycin (100 μg/ml).

### Isolation of Retinal Photoreceptors

Primary retinal cells were suspended in FACS Buffer, containing 0.5% bovine serum albumin (BSA), 2 mM EDTA-2H_2_O in FluoroBrite DMEM (Thermo Fisher Scientific, Japan), and were incubated with the anti-mouse CD73 Monoclonal Antibody (Thermo Fisher Scientific, Cat# MA5-15537, RRID: AB_10981671) for 1 h on ice as marker of retinal photoreceptor (Gagliardi et al., 2018). Cells were then washed with FACS Buffer for three times and centrifuged at 400 g for 5 min. Subsequently, cells were incubated with a secondary antibody, anti-mouse IgG microbeads (Miltenyi Biotech, Cat# 130-048-402, RRID: AB_244360) for 1 h on ice, and then washed three times again. Magnetic-activated cell sorting (MACS) was used to collect CD73(+) photoreceptor cells and CD73(-) non-photoreceptor cells according to the manufacturer’s instruction (Miltenyi Biotech, CA, United States), by loading cells on LS columns for magnetic separation. Collected cells were cultured in Eagle’s minimum essential medium (MEM) supplemented with penicillin (100 U/ml), streptomycin (100 μg/ml),10% heat−inactivated fetal bovine serum, 26 mM NaHCO_3_ and 25 mM HEPES following the protocol described by Maisto et al. (2017). After 48 h, primary retinal cells were characterized for opsin as marker of retinal photoreceptors and plated at density of 2.0 × 10^6^ cells for cm^2^. Then, cells were exposed for 96 h to physiological glucose concentration (normal glucose, 5 mM D-glucose); or to high glucose concentration (HG, 30 mM D-glucose) as a model of high glucose-induced acute retinal damage (Davinelli et al., 2017) or to high glucose medium stimulated with a single administration of 50 nM RvD1 (Tian et al., 2009) alone or combined with 20 μM Boc2, the FPR2 antagonist (Hughes et al., 2017). Finally, 24.5 mM D-mannitol was added to normal glucose medium, to obtain a final concentration of 30 mM as high osmotic control group (Trotta et al., 2019). This did not show evident alterations in photoreceptor physiology compared to cell exposed to normal glucose.

### Characterization of Retinal Photoreceptors

After fixation in 4% paraformaldehyde, as described by Maisto et al. (2017), photoreceptors were incubated with a primary monoclonal opsin antibody (Sigma-Aldrich, Cat# O4886, RRID: AB_260838) followed by Alexa Fluor^®^ 488 anti-mouse (Abcam, Cat# ab150113, RRID: AB_2576208) antibody. Cell nucleus was counterstained with Hoechst 33258 (Thermo Fisher Scientific, Cat# H3569, RRID: AB_2651133). Fluorescence intensity was quantified by LEICA software (RRID: SCR_016555)^1^. Positive opsin cells were counted and characterized accordingly to the previous published method (Maisto et al., 2017): green (opsin) positive cells/400 counted cells/field. Six different microscope fields for each preparation were analyzed and the opsin positive cells expressed accordingly to the equation = % cells^+opsin^/field. HUVEC cells were labeled with opsin as negative control.

### XTT Assay

3′-[1-phenylaminocarbonyl-3,4-tetrazolium]-bis(4-methoxy-6-nitro) benzene sulfonic acid hydrate (XTT; Roche, Cat# 11465015001) was used to assess cell viability, by measuring metabolic activity at 550 nm (Victor X5; Perkin Elmer). In brief, photoreceptors were seeded into microplates (96 wells) at a concentration of 4 × 10^3^ cells/well in 100 μl culture medium and treated as previously described in section Isolation of Retinal Photoreceptors. 0.3 mg/ml of the XTT final solution per well were added and cells were incubated for 6 h at 37°C and 6.5% CO2. The spectrophotometrical absorbance of the samples was measured at the wavelength of 450 nm, using a reference wavelength of 650 nm.

### Annexin V Assay

Apoptotic photoreceptors were detected using the fluorescein-conjugated Annexin V kit (Guava Nexin Reagent; Merck Millipore, Cat# 4500–0450) following the manufacturer’s instructions. Four cell populations were detected by using two separate dyes, Annexin V and the 7-aminoactinomycin D (7-AAD) dye on a Guava EasyCyte flow cytometer: non-apoptotic cells (Annexin V-negative and 7-AAD-negative); early apoptotic cells (Annexin V-positive and 7-AAD-negative); late-apoptotic (Annexin V-positive and 7-AAD-positive) and necrotic cells (Annexin V-positive and 7-AAD-positive) (Alessio et al., 2019).

### ROS Assay

2′7′-dichlorodihydrofluorescein diacetate (DCFH-DA; Santa Cruz Biotechnology, USACAS 4091–99-0) was used to assess ROS levels, proportional to fluorescence units (FU). Cells were incubated with 15 μM H_2_DCFDA for 15 min at 37°C in 5% CO_2_. Incubation with 100 μM H_2_O_2_ was considered as positive control. A fluorescence multiple reader (Victor X5; Perkin Elmer) was used to measure total intracellular ROS production at an excitation of 485 nm and an emission of 530 nm (Maisto et al., 2019).

### Protein Isolation

Photoreceptors were homogenized in RIPA buffer (Sigma-Aldrich, Cat# R0278) containing protease and phosphatase inhibitors. Samples were centrifuged at 12,000 rpm for 10 min at 4°C in order to remove any nucleic acids contaminants (Trotta et al., 2017). Then, protein levels were measured by using a Bio-Rad Protein Assay (Cat# 500−0006; Bio-Rad Laboratories), and the samples were normalized for protein content before Western Blotting and ELISA assays.

### NF-κB Determination

Following a 12% Sodium Dodecyl Sulfate–PolyAcrylamide Gel Electrophoresis (SDS–PAGE) and the protein electrotransfer onto a polyvinylidene difluoride (PVDF) membrane (Merck Millipore, Cat# IPFL10100), this was blocked, incubated with primary and secondary antibodies and then visualized according the protocol described by Trotta et al. (2017). Anti-actin (Santa Cruz Biotechnology, Cat# sc-8432, RRID: AB_626630), anti-NFκB p-65 (E379) (Abcam, Cat# ab32536, RRID: AB_776751), anti-mouse (Santa Cruz Biotechnology Cat# sc-2005, RRID: AB_631736) and anti-rabbit IgG-HRP antibodies (Santa Cruz Biotechnology Cat# sc-2004, RRID: AB_631746). Protein levels were quantified with densitometric analysis carried out with ImageJ software (ImageJ, RRID: SCR_003070).

### Exosomes Isolation and Characterization

Total Exosomes Isolation Reagent (Thermo Fisher Scientific, Cat# 4478359) was used in order to isolate exosomes released by photoreceptors cultured as previously described in section Isolation of Retinal Photoreceptors using 1% Exosome-depleted FBS (Thermo Fisher Scientific, Cat# 4478359) (Maisto et al., 2019). Exosome pellet was obtained through 20,000 × *g* centrifugation, stored at 4°C in Phosphate Buffered Saline (PBS 1x) and characterized with NanoSight NS300 following the manufacturer’s protocol (Malvern Instruments). Quantitative determination of the exosomal markers annexin A2 (Biocompare, Cat# OKEH05627) and flotillin-1 (Antibodies online, Cat# ABIN415105) (Emam et al., 2018) was performed in the exosomal pellet by using commercial ELISA kits. Electron microscopy for size and morphology evaluations (2000× magnification) was used, as described by Maisto et al. (2019).

### HUVEC Cell Culture and Angiogenesis Assay

Human Umbilical Vein Endothelial Cells (HUVEC; ATCC Cat# CRL-1730, RRID: CVCL_2959), were cultured in order to perform the angiogenesis assay in Matrigel Growth Factor Reduced (CORNING, Cat# 356238) (Maisto et al., 2019). Briefly, 1 × 10^5^ HUVEC cells/well in a 12 well plate were left alone or treated with primary retinal cells exosomes (333.3 μg of exosome protein/well) for 5 h. Specifically, HUVEC cells were seeded with standard medium containing exosomes released by photoreceptors growth in the following media: NG, HG, HG + RvD1 and HG + RvD1 + Boc2. Olympus CKX41 inverted microscope (Olympus), connected to an Olympus SC20 (Olympus) camera, was used to record the images. Two investigators blind of treatments randomly analyzed six images/well by using the Image Pro-Plus Software V.6 (Media Cybernetics), counting the number of nodes and tubes for each image per well and expressing them as a mean ± standard error of the mean (s.e.m.).

### HUVEC Antagomir Treatment

1 × 10^5^ HUVEC cells stimulated with an elevated glucose concentration (30 mM D-glucose) were transfected with anti-hsa-miR-20a-5p (Qiagen, Cat# MIN0000075), anti-hsa-miR-20a-3p (Qiagen, Cat# MIN0004493), anti-hsa-miR-20b (Qiagen. Cat# MIN0001413), anti-hsa-miR-106a-5p (Qiagen, Cat# MIN0000103) or negative control (Qiagen, Cat# 1027271) by using Lipofectamine 2000 reagent (Life Techonologies, Cat# 11668-027), according the manufacturer’s protocol. After 24 h from transfection, miRNA levels were monitored by qRT-PCR, by using the specific primers for hsa-miR-20a-5p (Qiagen, Cat# MS00003199), anti-hsa-miR-20a-3p (Qiagen, MS00009065), anti-hsa-miR-20b (Qiagen, MS00003206), and anti-hsa-miR-106a-5p (Qiagen. MS00008393). Transfected HUVEC cells were seeded in a 12 well plate with standard medium containing exosomes (333.3 μg of exosome protein/well) released after stimulation of transfected photoreceptors with NG, HG, HG + RvD1 or with HG + RvD1 + Boc2, as already described.

### Extraction and Expression Analysis of Intracellular or Exosomal miRNAs

Isolation of total RNA, including small RNAs, from photoreceptors or HUVEC cells was performed by using the MiRNeasy Mini kit (Qiagen, Cat# 74104). Exosomal miRNAs were collected from photoreceptor medium by using the exoRNeasy Serum/Plasma Maxi Kit (Qiagen, Cat# 77064), according to the Supplementary Protocol “Purification of exosomal RNA, including miRNA, from cell culture supernatants using the exoRNeasy Serum/Plasma Maxi Kit.” Syn-cel-miR-39 miScript miRNA Mimic 5 nM (Qiagen, Cat# MSY0000010) was added to monitor miRNA isolation (Trotta et al., 2017; Supplementary Figure S1). Reverse-Transcription Real Time PCR (qRT-PCR) was performed according the protocol described by Trotta et al. (2017), in order to assess mmu-miR-20a-3p, mmu-miR-20a-5p, mmu-miR-106a-5p, and mmu-miR-20b expression levels. MiScript II Reverse Transcription Kit (Qiagen, Cat# 218161), miScript SYBR Green PCR Master Mix (Qiagen, Cat# 218073), specific miScript primer Assays (Qiagen, Cat# MS00011473, MS00001309, MS00011039, MS00001316) and Ce-miR-39-5p as control (Qiagen, Cat# MS00080247) were used to carry out qRT-PCR reactions. ΔCt value for each miRNA was calculated as ΔCt = Ct _miRNA_ – Ct _Ce_miR–__39__–__5__p_, and then miRNas expression was then obtained as 2^–ΔCt^, as described by Trotta et al. (2017).

### RvD1, FPR2, and VEGF ELISA Assay

RvD1, FPR2 receptor, TNF-α, and VEGF levels were measured in photoreceptors by using: Resolvin D1 ELISA Kit (Cayman Chemical, Cat# 500380), Mouse Formyl Peptide Receptor 2 (FPR2) ELISA Kit (MyBiosource, Cat# MBS764510), Mouse Interleukin 18 (IL-18) ELISA Kit (Abcam, Cat# ab216165), Mouse VEGF Quantikine ELISA Kit (RandD System, Cat# MMV00) in retinal photoreceptors and Human VEGF ELISA Kit (MyoBiosurce, Cat# MBS355343) in HUVEC cells.

### Statistical Analysis

The results of each experiment are presented as the mean ± s.e.m. of three independent experimental settings, run in triplicate. One-way ANOVA, ANOVA for repeated measures and Bonferroni’s test were used to determine statistical significance among the groups with GraphPad Prism 6 software. Differences between experimental groups are considered statistically significant with the cutoff for *p*-values < 0.05.

## Results

### Retinal Photoreceptors Isolation and Characterization

**FIGURE 1:**
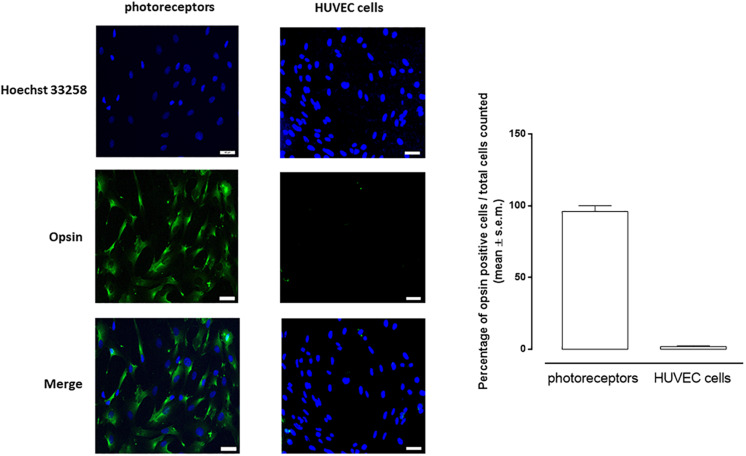
Representative images of immunocytochemistry of photoreceptors and HUVEC cells cultured in 5 mM D-glucose and labeled for opsin (green). Nuclei were stained with Hoechst 33258 (blue). Opsin-positive cell numbers are represented as white columns. The results are expressed as mean ± s.e.m. of the percentage of opsin positive cells/total cells counted for each microscopic field. Scale bar = 40 μM; magnification = 40×.

We successfully isolated from C57BL/6J mice CD73(+) retinal photoreceptors by MACS. Those cells were positive to opsin staining and yield 96 ± 4% cells, as calculated on total extracted cells (Figure 1).

### RvD1 Protects Cells From HG-Induced Damage

The observation of cell viability and death of photoreceptors during a time course of 96 h evidenced that cell viability time-dependently decreased when exposed to high glucose, reaching its maximum at 96 h (Supplementary Figures S2, S3). Therefore, this time point was for all the experiments.

**FIGURE 2:**
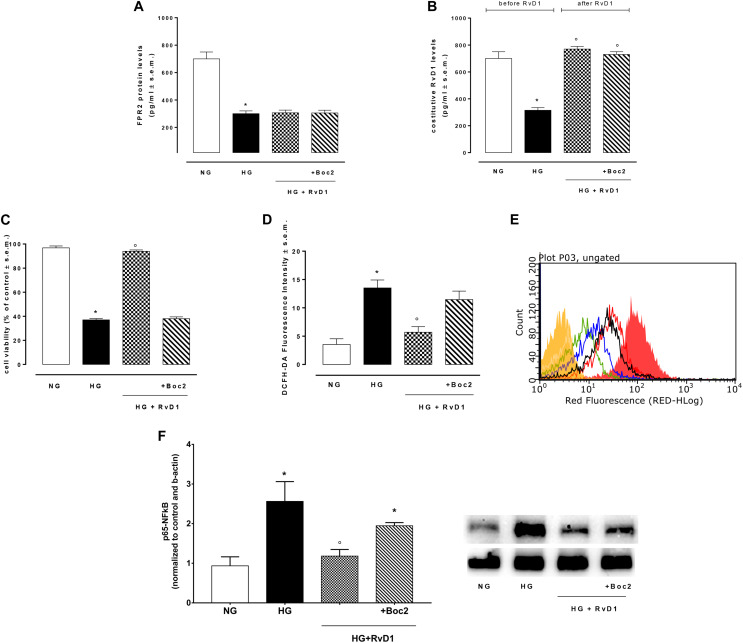
FPR2 and RvD1 levels, cell viability, ROS content, NF-kb protein expression. **(A)** ELISA detecting the levels of FPR2 receptor and **(B)** constitutive Resolvin D1 before and after RvD1 addition to photoreceptors exposed to high glucose; **(C)** XTT assay for determination of total cell number; **(D,E)** average intensity from DCFH-DA for total intracellular ROS levels compared to a negative control (yellow) and a positive control (fill red, 100 μM H_2_O_2_). Green = normal glucose; black = high glucose; blue = HG + RvD1 and red = HG + RvD1 + Boc2; **(F)** Western Blotting determination and representative images of NFκB protein levels into photoreceptors stimulated with normal glucose (5 mM D-glucose); high glucose (30 mM D-glucose); HG + RvD1 (RvD1, 50 nM); HG + RvD1 + Boc2 (20 μM). Values are expressed as mean ± s.e.m. of *n* = 9 values, obtained from the triplicates of three independent experiments. They were analyzed by one-way ANOVA followed by Bonferroni’s test for each panel, except for panel **(B)** were ANOVA for repeated measures was applied. NG, normal glucose; HG, high glucose; RvD1, Resolvin D1; Boc-2, selective FPR2 inhibitor. *P > 0.01 vs. NG; °*P* > 0.01 vs. HG.

Photoreceptors stimulated with 30 mM D-glucose showed a decreased expression of FPR2 receptor (Figure 2A), compared to control cells. Furthermore, photoreceptors growth in HG beard a lower RvD1 content compared to control levels (Figure 2B). Photoreceptors viability decreased of 59.8% after exposure to HG (30 mM D-glucose), compared to control cells (NG, 5 mM D-glucose). The treatment with 50 nM RvD1 significantly (*p* < 0.05) increased the percentage of viable cells 57%, compared to untreated cells exposed to HG (Figure 2C). Treatment with 20 μM Boc2 (HG + RvD1 + Boc2), the FPR2 antagonist, significantly attenuated the protective effect of RvD1 against the HG-induced damage (Figure 2C). Indeed, we tested the capability of RvD1 treatment as antioxidant in photoreceptors exposed to HG. After 96 h exposure to high glucose, cells produced significant (*p* < 0.01) higher ROS levels, compared to control (Figures 2D,E). RvD1 treatment (50 nM) significantly (*p* < 0.05) inhibited ROS production induced by HG (Figures 2D,E) while 20 μM of the FPR2 inhibitor Boc2 (HG + RvD1 + Boc2) abolished the RvD1 antioxidant effect (Figures 2D,E).

To confirm in our *in vitro* model the established anti-inflammatory activity of RvD1, we tested the levels of p65 NFκB in the photoreceptor culture. Levels of p65 NFκB were higher in cells growth in HG, compared to control cells (Figure 2F). RvD1 treatment decreased significantly p65 NFκB levels in photoreceptors exposed to HG. Accordingly, IL-18, the down-stream target of p65 NFκB, was reduced following RvD1 exposure (Supplementary Figure S4). Inhibition of FPR2 receptor with Boc2 (20 μM) abolished the anti-inflammatory activity of RvD1 (Figure 2F). Therefore, RvD1/FPR2 pathway is involved in modulation of inflammation and oxidative stress induced by HG.

### High Glucose Reduced Intracellular miRNAs Related to VEGF

**FIGURE 3:**
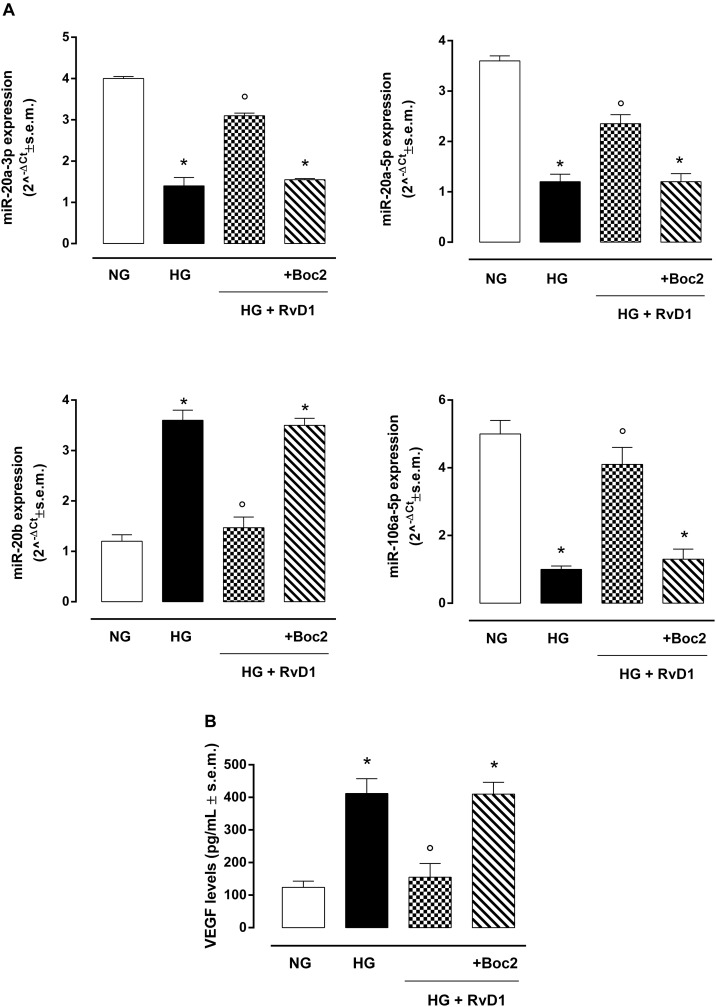
VEGF-related miRNAs and VEGF levels in photoreceptors. **(A)** qRT-PCR for cellular miRNAs miR-20a-3p, miR-20a-5p, miR-20b, miR-106a-5p expression determination and **(B)** ELISA test for VEGF levels in photoreceptors stimulated with normal glucose (5 mM D-glucose); high glucose (30 mM D-glucose); HG + RvD1 (RvD1, 50 nM); HG + RvD1 + Boc2 (20 μM). Values are expressed as mean ± s.e.m. of *n* = 9 values, obtained from the triplicates of three independent experiments. They were analyzed by one-way ANOVA followed by Bonferroni’s test for each panel. NG, normal glucose; HG, high glucose; RvD1, Resolvin D1; Boc2, selective FPR2 inhibitor. *P < 0.01 vs. NG; °*P* < 0.01 vs. HG.

The VEGF targeting miRNAs (miR-20a-3p, miR-20a-5p, and miR-106a-5p), named anti-angiogenic miRNAs, were significantly reduced in photoreceptor culture stimulated with HG compared to control cells (Figure 3A). Thus, VEGF levels were higher in cells exposed to HG compared to control (Figure 3B). Interestingly, miR-20b expression was increased after exposure to HG and decreased to control levels after treatment with RvD1. Expression of miR-20a-3p, miR-20a-5p, and miR-106a-5p, after RvD1 treatment, increased compared to cells exposed to HG. Boc2, significantly inhibited the effects of RvD1 by a significant decrease of miR-20a-3p and miR-106a levels. Interestingly, RvD1 decreased cellular expression of miR-20b, which was increased in HG exposed primary cells; Boc2 (HG + RvD1 + Boc2) abolished the effects of RvD1 on miR-20b expression, compared to RvD1 treated cells. Furthermore, Boc2 significantly decreased miR-20a-3p expression compared to RvD1 (Figure 3A). RvD1 effects on VEGF-related miRNA expression in HG cells were paralleled by a significant reduction in cell VEGF levels (Figure 3B). Interestingly, Boc2 (HG + RvD1 + Boc2) overall inhibited RvD1 anti-angiogenic effects by increasing VEGF levels (Figure 3B).

### Photoreceptors Release Exosomes While Exposed to HG

**FIGURE 4:**
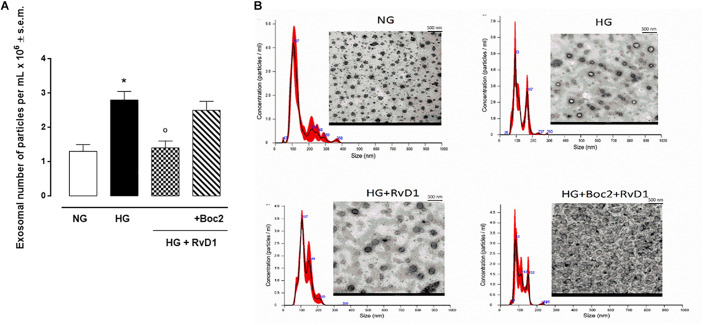
Nanosight and TEM of exosomes released from photoreceptors. Size-distribution of exosomes assessed using a Nanoparticle Tracking Analysis **(A)** and their release into the extracellular medium assessed by electron microscopy **(B)** in photoreceptors stimulated with normal glucose (5 mM D-glucose); high glucose (30 mM D-glucose); HG + RvD1 (RvD1, 50 nM); HG + RvD1 + Boc2 (20 μM). Scale bar 500 nm. Magnification 2000X. Values are expressed as mean ± s.e.m. of *n* = 9 values, obtained from the triplicates of three independent experiments. They were analyzed by one-way ANOVA followed by Bonferroni’s test for each panel. NG, normal glucose; HG, high glucose; RvD1, Resolvin D1; Boc-2, selective FPR2 inhibitor. *P < 0.01 vs. NG; °*P* < 0.01 vs. HG.

**FIGURE 5:**
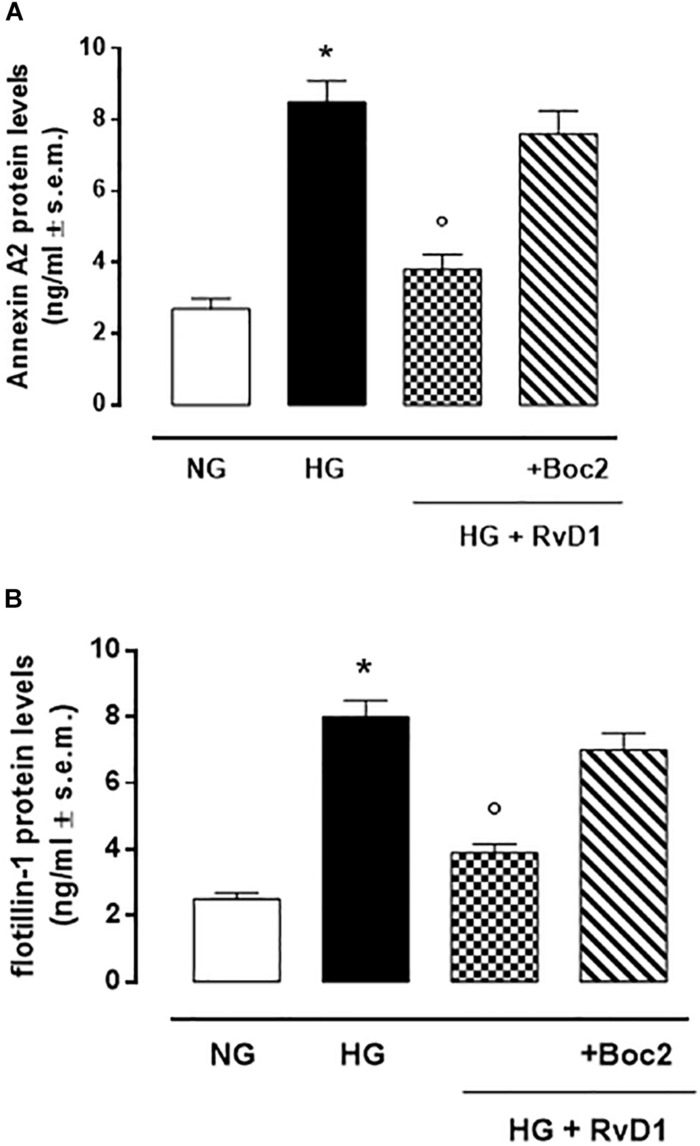
Annexin A2 **(A)** and Flottilin-1 **(B)** labeling of exosomes released from primary photoreceptors stimulated with normal glucose (5 mM D-glucose); high glucose (30 mM D-glucose); HG + RvD1 (RvD1, 50 nM); HG + RvD1 + Boc2 (20 μM). Values are expressed as mean ± s.e.m. of *n* = 9 values, obtained from the triplicates of three independent experiments. They were analyzed by one-way ANOVA followed by Bonferroni’s test for each panel. NG, normal glucose; HG, high glucose; RvD1, Resolvin D1; Boc-2, selective FPR2 inhibitor. *P < 0.01 vs. NG; °*P* < 0.01 vs. HG.

We found that exposure to photoreceptors to high glucose e induced release of small vesicles that were exosomes as assessed by Nanoparticle Tracking Analysis (Figure 4A) and Transmission Electron Microscopy imaging (Figure 4B). Furthermore, exosomes expressed in their surface specific validated markers annexin A2 and flotillin-1 (Figures 5A,B). Photoreceptors exposure to different media modified exosome markers expression patterns. HG increased the number of released exosomes (+115.3% increase) (Figures 4A,B). High glucose exposure increased the annexin A2 and flotillin-1 protein levels to + 222.2% and + 220%, respectively (Figures 5A,B). Interestingly, photoreceptor culture treated with RvD1 released significantly less exosomes, about 50% decrease, compared to non-treated cells exposed to HG. Boc2 abolished the RvD1 effect, leading to exosome number not dissimilar to exosome levels released by cells exposed to HG (Figures 4, 5).

### RvD1 Treatment Modified miRNA and VEGF Content in Exosomes Released by Photoreceptors

**FIGURE 6:**
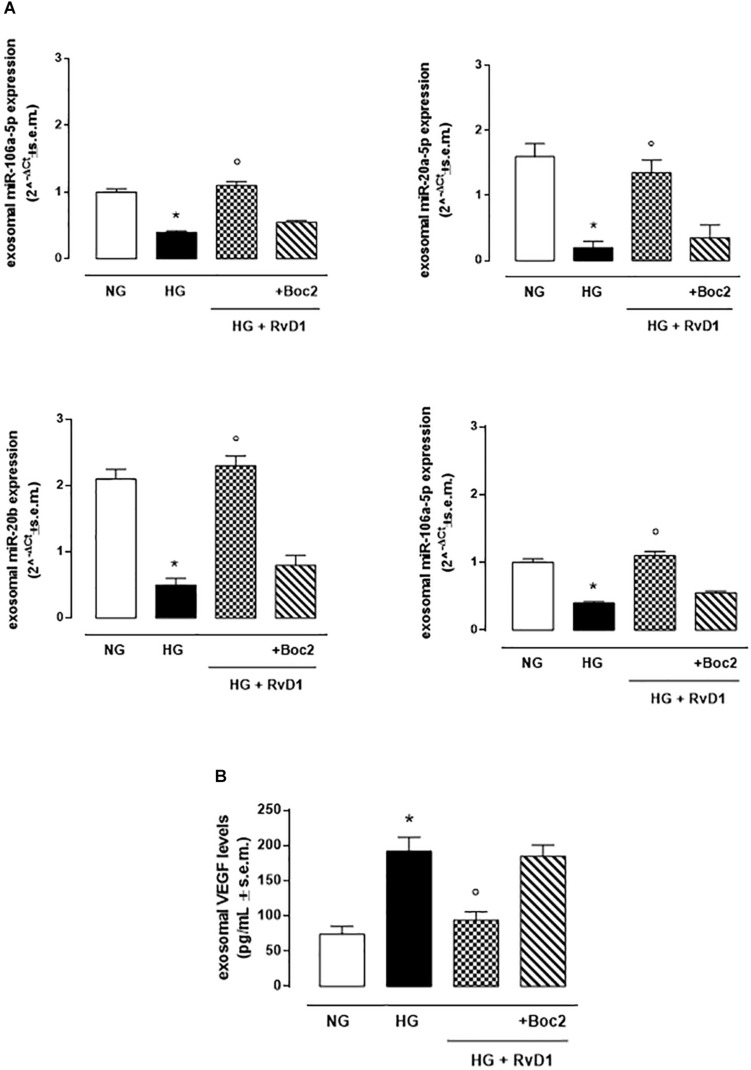
Exosomal miRNAs and VEGF levels. **(A)**qRT-PCR for miR-20a-5p, miR-20a-3p, miR-20b, miR-106a-5p expression and **(B)** ELISA test for VEGF levels from exosomes released after stimulation of photoreceptors with normal glucose (5 mM D-glucose); high glucose (30 mM D-glucose); HG + RvD1 (RvD1, 50 nM); HG + RvD1 + Boc2 (20 μM). Values are expressed as mean ± s.e.m. of *n* = 9 values, obtained from the triplicates of three independent experiments. They were analyzed by one-way ANOVA followed by Bonferroni’s test for each panel. NG, normal glucose; HG, high glucose; RvD1, Resolvin D1; Boc-2, selective FPR2 inhibitor. *P < 0.01 vs. NG; ° *P* < 0.01 vs. HG.

**FIGURE 7:**
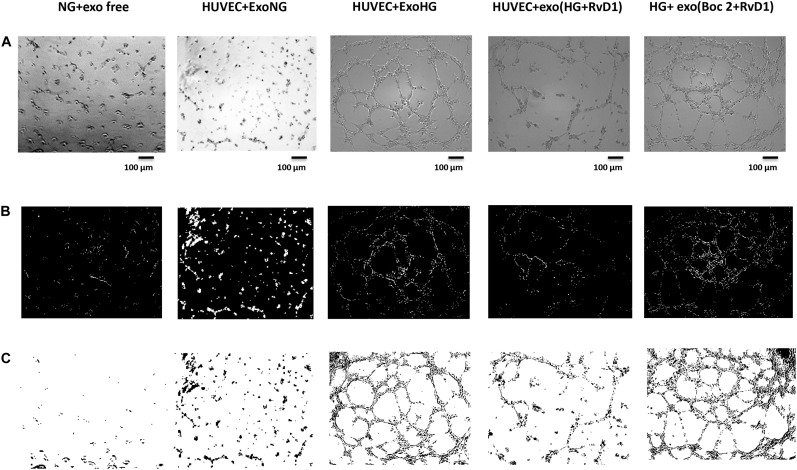
Representative images of the tubular structures from non-transfected HUVEC cells. **(A)** Matrigel natural views, **(B)** dark field Matrigel views and **(C)** Matrigel graphical images of HUVEC cells grown in normal glucose (NG, 5 mM) seeded with: exosome-free medium (NG-exofree); standard medium containing exosomes released after stimulation of photoreceptors with Normal Glucose (NG, 5 mM) (NG + exoNG); standard medium containing exosomes released after stimulation of photoreceptors with High Glucose (HG, 35 mM) (NG + exoHG); standard medium containing exosomes released after stimulation of photoreceptors with HG + RvD1 (50 nM) (NG + exoHG-RvD1); standard medium containing exosomes released after stimulation of photoreceptors with HG + RvD1 + Boc2 (20 μM) (NG + exoHG-RvD1 + Boc2). Scale bar 100 μm. Magnification 100X.

High glucose (30 mM) exposure increased the number of VEGF-marked exosomes released by photoreceptors (Figure 6B). These contained significantly downregulated anti-angiogenic miRNAs, compared to exosomes released by cells growth in control medium (glucose 5 mM) (Figure 6A). RvD1 decreased the number of VEGF-marked exosomes (Figure 7B), while it increased the expression of anti-angiogenic miRNAs (Figure 6A). The upregulation of miR-20a-3p, miR-20a-5p, miR-106, and miR-20b, was counteracted by the Boc 2, FPR2 antagonist, treatment (Figure 6A).

### Exosomes Released by Photoreceptors Treated With RvD1, Showed a Decreased Pro-angiogenic Potential, Compared to Exosomes Released by High-Glucose Treated Cells

**FIGURE 8:**
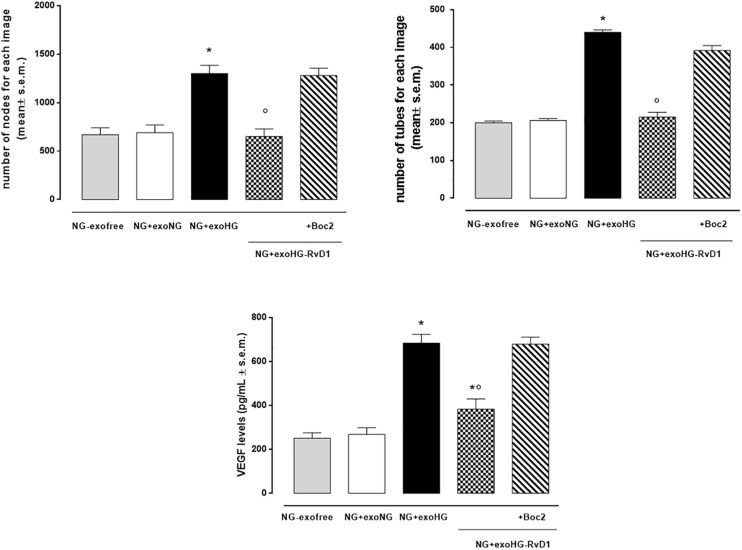
Bar graphs showing the mean number of nodes formation and the mean number of tubes formation for each treatment, along with VEGF levels in non-transfected HUVEC cells grown in normal glucose (NG, 5 mM) seeded with: exosome-free medium (NG-exofree); standard medium containing exosomes released after stimulation of photoreceptors with Normal Glucose (NG, 5 mM) (NG + exoNG); standard medium containing exosomes released after stimulation of photoreceptors with High Glucose (HG, 35 mM) (NG + exoHG); standard medium containing exosomes released after stimulation of photoreceptors with HG + RvD1 (50 nM) (NG + exoHG-RvD1); standard medium containing exosomes released after stimulation of photoreceptors with HG + RvD1 + Boc2 (20 μM) (NG + exoHG-RvD1 + Boc2). The values are expressed as the mean ± s.e.m. of *n* = 9 values obtained from the triplicates of three independent experiments. They were analyzed by one-way ANOVA followed by Bonferroni’s test for each panel. *P < 0.01 vs. NG + exoNG and °*P* < 0.01 vs. NG + exoHG.

**FIGURE 9:**
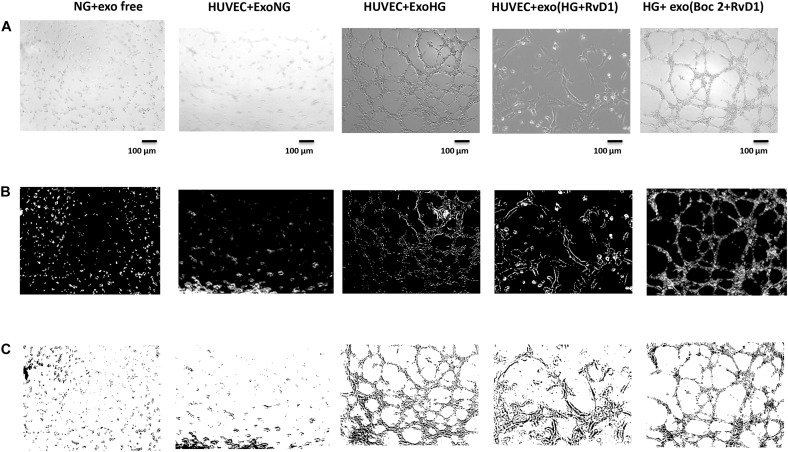
Representative images of the tubular structures and node formation from transfected HUVEC cells. **(A)** Matrigel natural views, **(B)** dark field Matrigel views, and **(C)** Matrigel graphical images of HUVEC cells grown in normal glucose (NG, 5 mM) after the silencing of miR-20a-5p, miR-20a-3p, miR-20b, and miR-106a-5p in these cells. Cell seeded with exosome-free medium (NG-exofree); standard medium containing exosomes released after stimulation of primary cells with Normal Glucose (NG, 5 mM) (NG + exoNG); standard medium containing exosomes released after stimulation of photoreceptors with High Glucose (HG, 35 mM) (NG + exoHG); standard medium containing exosomes released after stimulation of photoreceptors with HG + RvD1 (50 nM) (NG + exoHG-RvD1); standard medium containing exosomes released after stimulation of photoreceptors with HG + RvD1 + Boc2 (20 μM) (NG + exoHG-RvD1 + Boc2). Scale bar 100 μm. Magnification 100X.

**FIGURE 10:**
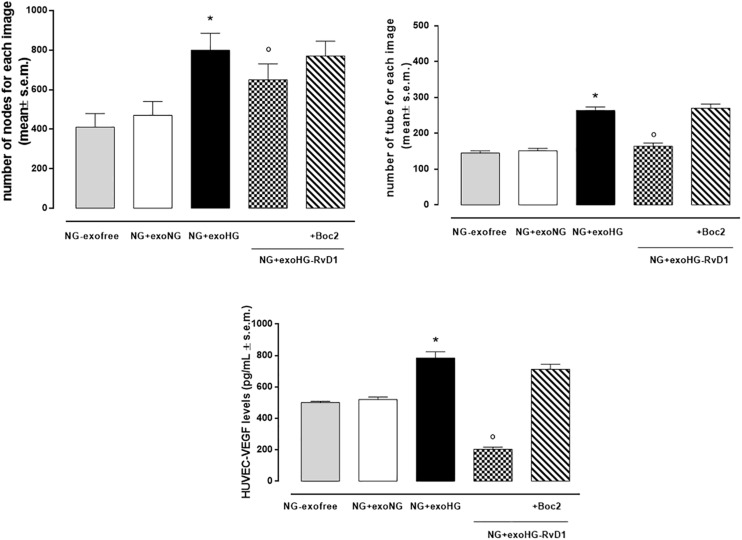
Bar graphs showing the mean number of nodes formation and the mean number of tubes formation for each treatment, along with VEGF levels in transfected HUVEC cells grown in normal glucose (NG, 5 mM) after the silencing of miR-20a-5p, miR-20a-3p, miR-20b, and miR-106a-5p in these cells. Cell seeded with exosome-free medium (NG-exofree); standard medium containing exosomes released after stimulation of photoreceptors with Normal Glucose (NG, 5 mM) (NG + exoNG); standard medium containing exosomes released after stimulation of photoreceptors with High Glucose (HG, 35 mM) (NG + exoHG); standard medium containing exosomes released after stimulation of photoreceptors with HG + RvD1 (50 nM) (NG + exoHG-RvD1); standard medium containing exosomes released after stimulation of photoreceptors with HG + RvD1 + Boc2 (20 μM) (NG + exoHG-RvD1 + Boc2). The values are expressed as the mean ± s.e.m. of *n* = 9 values obtained from the triplicates of three independent experiments. They were analyzed by one-way ANOVA followed by Bonferroni’s test for each panel. The significance levels are expressed as *P < 0.01 vs. NG + exoNG and °*P* < 0.01 vs. NG + exoHG.

By means of the angiogenesis assay, we confirmed that high glucose released exosomes, with a high VEGF content, promote node and tube formation in HUVECs (Figures 7, 8). RvD1 modified the number of exosomes released by treated cells, abolishing the pro-angiogenic potential of the exosomes released by cells exposed to HG, possibly by affecting their content. In order to test this latter hypothesis, we treated HUVECs with miR-20a-3p, miR-20a-5p, miR-106a-5p, and miR-20b antagomirs and exosomes released by primary cells exposed to HG + RvD1. Antagomirs caused a significantly reduction of HUVEC miRNAs levels compared to negative control transfected cells (Supplementary Figure S5) and an increase of VEGF content in HUVEC cells (with a value of 500 ± 9 pg/ml), with respect to cells transfected with negative control mimic (9 ± 1.5 pg/ml). HUVEC cells exposed to exosomes from photoreceptors treated with RvD1 showed the lowest VEGF content and number of tubes formed, respect to HUVEC cells exposed to exosomes released by photoreceptors treated with high glucose (Figures 9, 10).

## Discussion

Diabetic retinopathy (DR) has been regarded as a vascular disorder for many years, even inflammation has gained a central role in the pathophysiology of the disease (Rübsam et al., 2018). Currently, corticosteroids and anti-Vascular Endothelial Growth Factor (VEGF) are the standard therapeutic approach for the management of DR, particularly for proliferative DR and diabetic macular edema.

Besides a variety of pre-clinical and clinical studies, no drugs have been approved for treatment of non-proliferative diabetic retinopathy (NPDR) so far. Recently, treatment with ω3-PUFA and pro-resolving lipid mediators has been investigated in experimental studies (Das, 2013; Wang and Daggy, 2017; de Gaetano et al., 2018) as novel therapeutic strategy in DR. Among the pro-resolving mediators, resolvin D1 (RvD1) has emerged as the most promising alternative anti-inflammatory drug for treatment of DR. Derived from the ω3-PUFA docosahexaenoic acid (DHA) (Serhan et al., 2008), RvD1 is a potent lipid mediator able to promote inflammatory resolution in several inflammatory eye diseases, including endotoxic-induced uveitis (Rossi et al., 2012, 2015a,b; Calder, 2015). RvD1 improved the immune responses in a model of chronic allergic eye disease (Kaye et al., 2019; Saban et al., 2019), and promoted corneal epithelial wound healing in diabetic mice (Zhang et al., 2018). Moreover, RvD1 inhibited the leucocyte migration through choroid-retinal endothelial cells monolayer after interleukin 1β (IL-1β) stimulation (Tian et al., 2009). Recently, RvD1 has been shown to exert a suppressive action of inflammationin *in vitro* (Shi et al., 2017) and *in vivo* model of DR (Yin et al., 2017).

RvD1 endogenous levels, along with the expression of its receptor formyl peptide receptor 2 (ALX/FPR2), were reduced in diabetic retina (Shi et al., 2017). Noteworthy, RvD1 exogenous administration inhibits the inflammatory response through the inactivation of NF-kB signaling pathway (Yin et al., 2017). This last evidence in line with the results obtained here since RVD1 decreases the expression of NF-kB into primary retinal cells exposed to high glucose concentrations. Interestingly, this transcription factor over-activated by hyperglycemia-induced oxidative stress leads to altered expression of several gene targets. Among these increased VEGF (Maisto et al., 2019) and decrease of related inhibitory miRNAs are demonstrated to result in cells damage and modified angiogenesis in diabetic retinopathy (He and King, 2004; Kowluru and Chan, 2007; Patel and Santani, 2009; Kitada et al., 2010; Szade et al., 2015; Suryavanshi and Kulkarni, 2017; Yin et al., 2017; Platania et al., 2019).

microRNAs (miRNAs), small non-coding RNA molecules that regulate gene expression at post-transcriptional level, have recently emerged as essential regulators for the orchestration of retina homeostasis and angiogenesis (Nunes et al., 2015; Platania et al., 2019). Although we do not know which ones are more important at mediating the effects or whether their expression is modulated differentially, here it is depicted a down-regulation of four intracellular VEGF-related inhibitory miRNAs involved in the control of neovasculature. They are miR-20a-3p, miR-20a-5p, miR-106a-5p, and miR-20b, decreased in their normal expression by exposure of primary retinal photoreceptors to a high glucose charge. This leads to consequent increase of the protein VEGF. These data are in agreement with a previous report by Nunes et al. (2015) which validated the down-regulation of these miRNAs in early diabetes and retinal neovascularization *in vivo*, together with locally increased VEGF (Nunes et al., 2015). Additionally, miR-20a was predicted to act as a tumor suppressor and was found significantly down-regulated in the serum of advanced breast cancer patients (Jurkovicova et al., 2017). Moreover, miR-20b was able to regulate VEGF expression in a rat model of DR, both *in vitro* and *in vivo* (Qin et al., 2016).

In the present paper is also demonstrated that the specialized pro-resolving lipid mediator RvD1 affected the miRNAs biogenesis and promoted restoration of the normal intracellular content of these miRNAs, possibly via the canonical RNA interference pathway of Drosha, DiGeorge syndrome critical region 8 (DGCR8) and Dicer (Pong and Gullerova, 2018), deserving, however, more detailed investigation.

A recent study by Platania et al. (2019) revealed that the objective of these four miRNAs is to control the levels of VEGF in the same cell that produces both these factors, probably acting by autocrine action. This autocrine action, however, may not be the only one exerted by the miRNAs since it is demonstrated here for the first time that primary photoreceptors release exosomes into the extracellular space, under high glucose stimulus, that contain VEGF and VEGF-related miR-20a-3p, miR-20a-5p, miR-106a-5p, and miR-20b. It would be interestingly to potentially knock-down or over-express each microRNA to see which one is having the effects, but it deserves deeper investigation.

Interestingly, the anti-angiogenic miRNAs were downregulated also in exosomes released by primary endothelial cells exposed to high glucose levels. Indeed, ocular tissues during diabetes, even in early phases, could release exosomes with altered expression patterns of miRNAs, working as paracrine mediators promoting expression of VEGF in other recipient tissues. Our data, confirmed that circulating miRNAs, through cargo exosomes, are not only biomarkers but also hormone-like molecules, capable of information transfer activity triggering biological responses in recipient cells (Yuan et al., 2009).

Considering the miRNA-containing exosomes as potential molecules transferring informations that trig biological responses in receiving cells (Yuan et al., 2009), exosomes from photoreceptors cultures treated with the RvD1 and HUVEC cells as receiving cells were used in a setting to study the control exerted by the pathway RvD1-miRNA-VEGF-exosomes on the high glucose-induced neovascularization. For this, miR- 20a-, miR- 20b-, and miR-106a-containing exosomes released after treatment of high glucose-stimulated photoreceptors with RvD1 were used in a setting of high glucose-stimulated HUVEC cells. Once the exosomes extracted from the medium of photoreceptor culture were added to the HUVEC cell culture medium a decrease of node and tube formation was noted in these cells. This accounted for reduced transfer of VEGF by exosomes from photoreceptors and to the reduced *de novo* synthesis of VEGF in HUVEC by an inhibitory action on VEGF gene by the miRNAs transferred. Indeed, by silencing miR-20a-3p, miR-20a-5p, miR-106a-5p, and miR-20b in HUVEC cells the amount of VEGF protein formed by these cells increased. Moreover, the formation of nodes and tubes was increased for the same number of exosomes transferred. The mechanism by which RvD1 might be affecting the number and the cargo of exosome for microRNA content warrants further investigation at this time. However, the hypothesis formulated for the RvD1 parent compound DHA in initial studies by Hannafon et al. (2015) could apply for the metabolic bioproduct of docosahexaenoic acid (DHA) RvD1. This is based on a regulation by DHA of the exosome secretion and microRNA encapsulation through ceramide-dependent process into the producing cells. In fact, it well known that exosomes contain ceramide and their formation and release is inhibited by neutral sphingomyelinase inhibitors (Hannafon et al., 2015). Similarly, over-expression of sphingomyelinase increases extracellular levels of microRNAs, whereas treatment with a sphingomyelinase inhibitor reduces the miRNAs secretion (Hannafon et al., 2015). It is not clear, however, how VEGF-containing exosomes can activate cell-surface type 2 VEGF receptors (VEGFR2). However, exosomes influence the recipient cell as paracrine effectors, containing genetic material, proteins and factors essential for cell-cell communication, which can be transferred from one cell to another (Maisto et al., 2019). In conclusion, RvD1 by modulating the expression of ROS-induced NF-kB signaling causes changes of the intracellular miRNAs miR-20a-3p, miR-20a-5p, miR-106a-5p, and miR-20b of primary retinal photoreceptors stimulated with high glucose, and the release of these miRNAs-containing exosomes. This possibly underlying a causative role of these VEGF- related miRNAs for the neovascularization of the retina *in vivo* and a new tool for RvD1 to control the pathway miRNA-exosomes-cell proliferation in diabetic retinopathy, by targeting exosome secretion and their transmission content. The FPR2 receptor represents a bridge between all the RvD1 actions on the inflammation, gene regulation and microvascular damages in diabetic retinopathy rats (Recchiuti et al., 2011; Recchiuti and Serhan, 2012; Yin et al., 2017; Bisicchia et al., 2018). Therefore, the block of FPR2 receptor is crucial in order to attenuate the action of RvD1 on VEGF and VEGF-related miRNAs and control neoangiogenesis (Supplementary Figure S6).

## Data Availability Statement

All datasets generated for this study are included in the article/Supplementary Material.

## Ethics Statement

The Animal Ethics Committee of University of Campania “Luigi Vanvitelli” approved all the experimental procedures (Protocol Number 2108).

## Author Contributions

RM, MC, and CP conceived and designed the experiments. RM, MC, and FP performed the experiments. GC, MG, and RA analyzed the data. AH, JB, and SI contributed reagents, materials and analysis tool. MD’A and CB wrote the manuscript.

## Conflict of Interest

The authors declare that the research was conducted in the absence of any commercial or financial relationships that could be construed as a potential conflict of interest.

## Footnotes

1 https://www.leica-microsystems.com/products/microscope-software/details/product/leica-application-suite/
