# Corrigendum: Resolvin D1 Modulates the Intracellular VEGF-Related miRNAs of Retinal Photoreceptors Challenged With High Glucose

**DOI:** 10.3389/fphar.2020.00871

**Published:** 2020-06-16

**Authors:** Rosa Maisto, Maria Consiglia Trotta, Francesco Petrillo, Sara Izzo, Giovanna Cuomo, Roberto Alfano, Anca Hermenean, Jorge Miquel Barcia, Marilena Galdiero, Chiara Bianca Maria Platania, Claudio Bucolo, Michele D’Amico

**Affiliations:** ^1^Section of Pharmacology, Department of Experimental Medicine, University of Campania “Luigi Vanvitelli”, Naples, Italy; ^2^Department of Experimental Medicine, University of Campania “Luigi Vanvitelli”, Naples, Italy; ^3^Multidisciplinary Department of Surgical and Dental Specialties, University of Campania “Luigi Vanvitelli”, Naples, Italy; ^4^Department of Precision Medicine, University of Campania “Luigi Vanvitelli”, Naples, Italy; ^5^Department of Advanced Medical and Surgical Sciences, University of Campania “Luigi Vanvitelli”, Naples, Italy; ^6^Institute of Life Sciences, VasileGoldis Western University of Arad, Arad, Romania; ^7^School of Medicine, Catholic University of Valencia “Saint Vicente Martir”, Valencia, Spain; ^8^Department of Biomedical and Biotechnological Sciences, School of Medicine, University of Catania, Catania, Italy

**Keywords:** retinal photoreceptors, exosomes, miRNAs, resolvin D1, VEGF

In the original article, there was a mistake in **Figure 2F** as published. The wrong image was included due to the incorrect labeling of a file. The correct **Figure 2** appears below.

**Figure 2:**
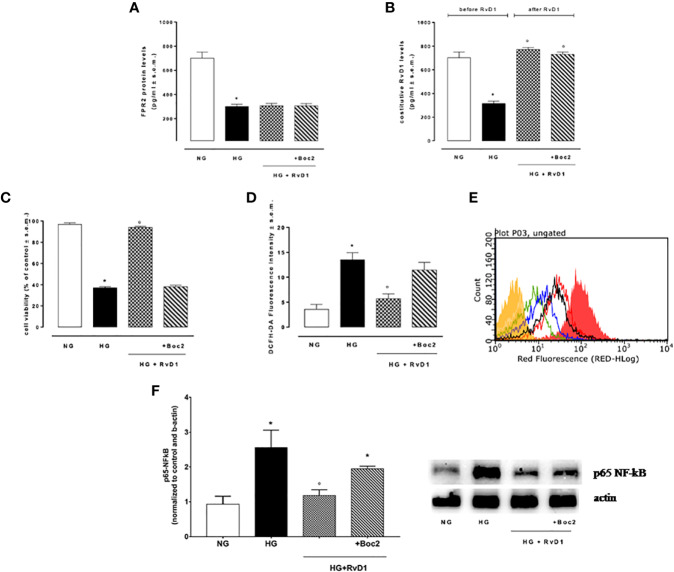
FPR2 and RvD1 levels, cell viability, ROS content, NF-kb protein expression. **(A)** ELISA detecting the levels of FPR2 receptor and **(B)** constitutive Resolvin D1 before and after RvD1 addition to photoreceptors exposed to high glucose; **(C)** XTT assay for determination of total cell number; **(D, E)** average intensity from DCFH-DA for total intracellular ROS levels compared to a negative control (yellow) and a positive control (fill red, 100 μM H2O2). Green = normal glucose; black = high glucose; blue = HG + RvD1 and red = HG + RvD1 + Boc2; **(F)** Western Blotting determination and representative images of NFκB protein levels into photoreceptors stimulated with normal glucose (5 mM D-glucose); high glucose (30 mM D-glucose); HG + RvD1 (RvD1, 50 nM); HG + RvD1 + Boc2 (20 μM). Values are expressed as mean ± s.e.m. of n = 9 values, obtained from the triplicates of three independent experiments. They were analyzed by one-way ANOVA followed by Bonferroni’s test for each panel, except for panel **(B)** were ANOVA for repeated measures was applied. NG, normal glucose; HG, high glucose; RvD1, Resolvin D1; Boc-2, selective FPR2 inhibitor. *P > 0.01 vs. NG; °P > 0.01 vs. HG.

In addition, there was a mistake in **Figure 7**. Incorrect panels were included in **Figures 7B, C**. The corrected **Figure 7** appears below.

**Figure 7:**
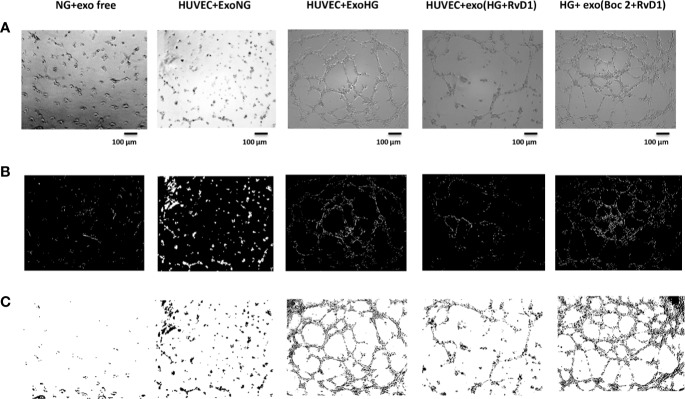
Representative images of the tubular structures from non-transfected HUVEC cells. **(A)** Matrigel natural views, **(B)** dark field Matrigel views and **(C)** Matrigel graphical images of HUVEC cells grown in normal glucose (NG, 5 mM) seeded with: exosome-free medium (NG-exofree); standard medium containing exosomes released after stimulation of photoreceptors with Normal Glucose (NG, 5 mM) (NG + exoNG); standard medium containing exosomes released after stimulation of photoreceptors with High Glucose (HG, 35 mM) (NG + exoHG); standard medium containing exosomes released after stimulation of photoreceptors with HG + RvD1 (50 nM) (NG + exoHG-RvD1); standard medium containing exosomes released after stimulation of photoreceptors with HG + RvD1 + Boc2 (20 μM) (NG + exoHG-RvD1 + Boc2). Scale bar 100 μm. Magnification 100X.

Finally, there was a mistake in **Figure 9** where the first two and last columns were mislabelled. The corrected **Figure 9** appears below.

**Figure 9:**
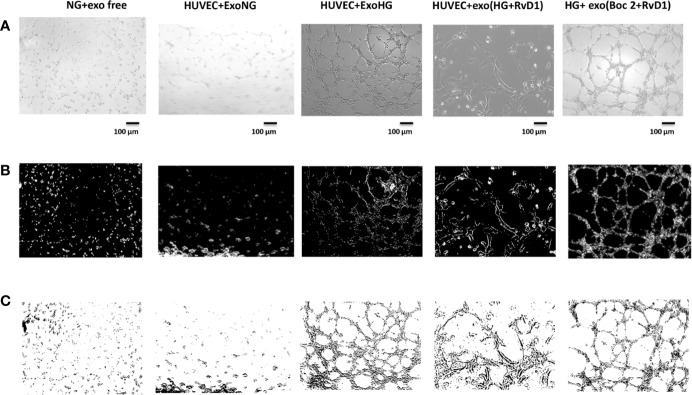
Representative images of the tubular structures and node formation from transfected HUVEC cells. **(A)** Matrigel natural views, **(B)** dark field Matrigel views, and **(C)** Matrigel graphical images of HUVEC cells grown in normal glucose (NG, 5 mM) after the silencing of miR-20a-5p, miR-20a-3p, miR-20b, and miR-106a-5p in these cells. Cell seeded with exosome-free medium (NG-exofree); standard medium containing exosomes released after stimulation of primary cells with Normal Glucose (NG, 5 mM) (NG + exoNG); standard medium containing exosomes released after stimulation of photoreceptors with High Glucose (HG, 35 mM) (NG + exoHG); standard medium containing exosomes released after stimulation of photoreceptors with HG + RvD1 (50 nM) (NG + exoHG-RvD1); standard medium containing exosomes released after stimulation of photoreceptors with HG + RvD1 + Boc2 (20 μM) (NG + exoHG-RvD1 + Boc2). Scale bar 100 μm. Magnification 100X.

The authors apologize for these errors and state that these do not change the scientific meaning and conclusions of the article in any way. The original article has been updated.

