# Supplementary material for: Resolvin D1 Modulates the Intracellular VEGF-Related miRNAs of Retinal Photoreceptors Challenged With High Glucose
Source: Front Pharmacol. 2020 Mar 6;11:235. doi: 10.3389/fphar.2020.00235 (PMC7069219; doi:10.3389/fphar.2020.00235)
Supplement: Supplementary file 1 [file Data_Sheet_1.docx]

**Figure S1**

|  | **NG** | **HG** | **HG+RvD1** | **HG+RvD1+Boc2** |
| --- | --- | --- | --- | --- |
| **Syn-Cel-miR-39 (Ct)** | 19.6 ± 1.3 | 19.8 ± 0.9 | 19.7 ± 1,2 | 19.6 ± 1,1 |

**Figure S1.** Ct values of Syn-Cel-miR-39 detected by qRT-PCR in primary retinal cells stimulated with normal glucose (5 mM D-glucose); high glucose (30 mM D-glucose); HG+RvD1 (RvD1, 50 nM); HG+RvD1+Boc2 (20 µM). Values are expressed as mean ± s.e.m. of n = 9 values, obtained from the triplicates of three independent experiments. They were analyzed by one-way ANOVA followed by Bonferroni test’s.

**Figure S2**

**
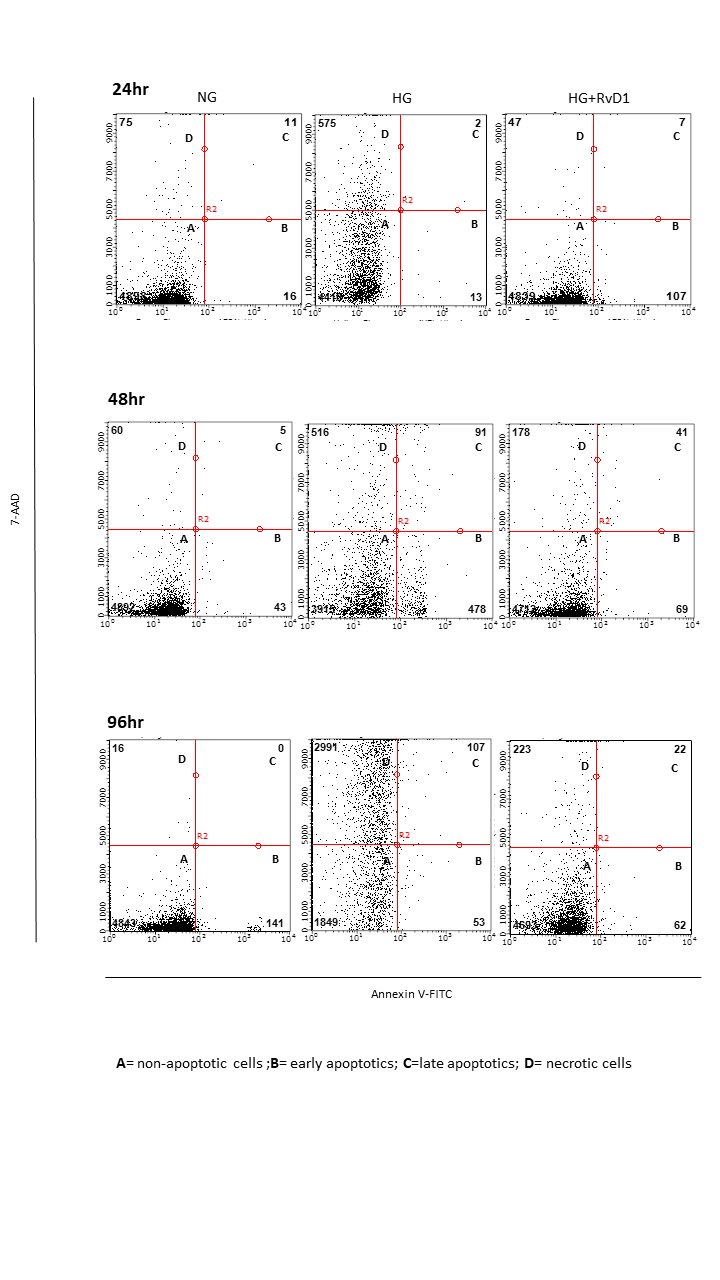
**

**Figure S2: Representative scatter plots of apoptotic cell population.** Apoptotic cells were detected at 24, 48 and 96 hours using the Annexin V/7-AAD staining. The spectrum of apoptotic and non‐apoptotic cells are identified by the binding of Annexin V to phosphatidylserine on the external membrane of apoptotic cells and the staining of 7‐aminoactinomycin D (7‐AAD) in late‐stage apoptotic and dead cells. **(A****)** non‐apoptotic cells (Annexin V‐negative and 7‐AAD‐negative); **(B)** early apoptotic cells (Annexin V‐positive and 7‐AAD‐negative); **(C)** late‐apoptotic (Annexin V‐positive and 7‐AAD‐positive) and **(D)** necrotic cells (Annexin V‐positive and 7‐AAD‐positive). NG = normal glucose (5 mM D-glucose); HG = high glucose (30 mM D-glucose); HG+RvD1 = HG cells + Resolvin D1, 50 nM.

**Figure S3**

**
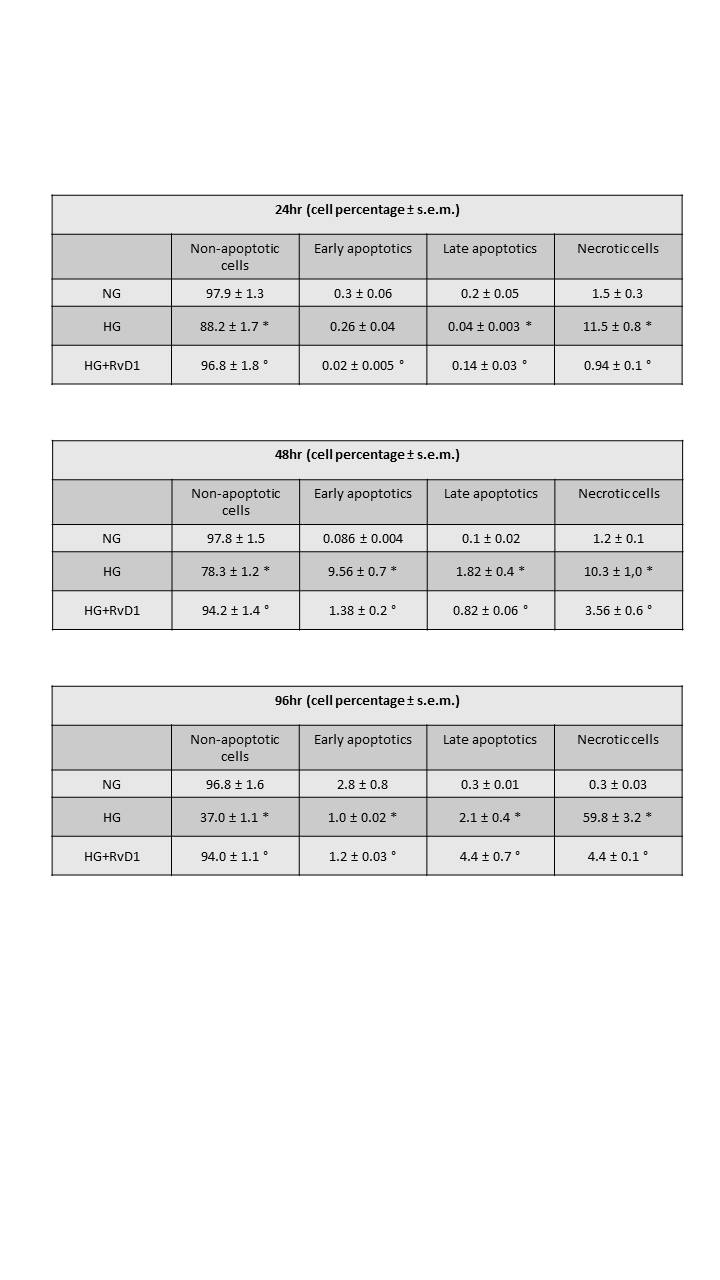
**

**Figure S3: Values of non-apoptotic, early apoptotic, late apoptotic and necrotic cells.** Apoptotic cell population at 24, 48 and 98 hours determined by Annexin V/7-AAD staining in in primary retinal cells stimulated with normal glucose (5 mM D-glucose); high glucose (30 mM D-glucose); HG+RvD1 (RvD1, 50 nM). Values are expressed as mean ± s.e.m. of n = 9 values, obtained from the triplicates of three independent experiments. NG = normal glucose; HG = high glucose; RvD1 = Resolvin D1. *P<0.01 vs. NG; °P<0.01 vs. HG. They were analyzed by one-way ANOVA followed by Bonferroni test’s.

**Figure S4**

**Figure S4:** **ELISA test for IL-18 levels** from photoreceptors exposed to normal glucose (5 mM D-glucose); high glucose (30 mM D-glucose); HG+RvD1 (RvD1, 50 nM); HG+RvD1+Boc2 (20 µM). Values are expressed as mean ± s.e.m. of n = 9 values, obtained from the triplicates of three independent experiments. They were analyzed by one-way ANOVA followed by Bonferroni test’s for each panel. NG = normal glucose; HG = high glucose; RvD1 = Resolvin D1; Boc-2 = selective FPR2 inhibitor. *P<0.01 vs. NG; °P<0.01 vs. HG.

**Figure S5**

**
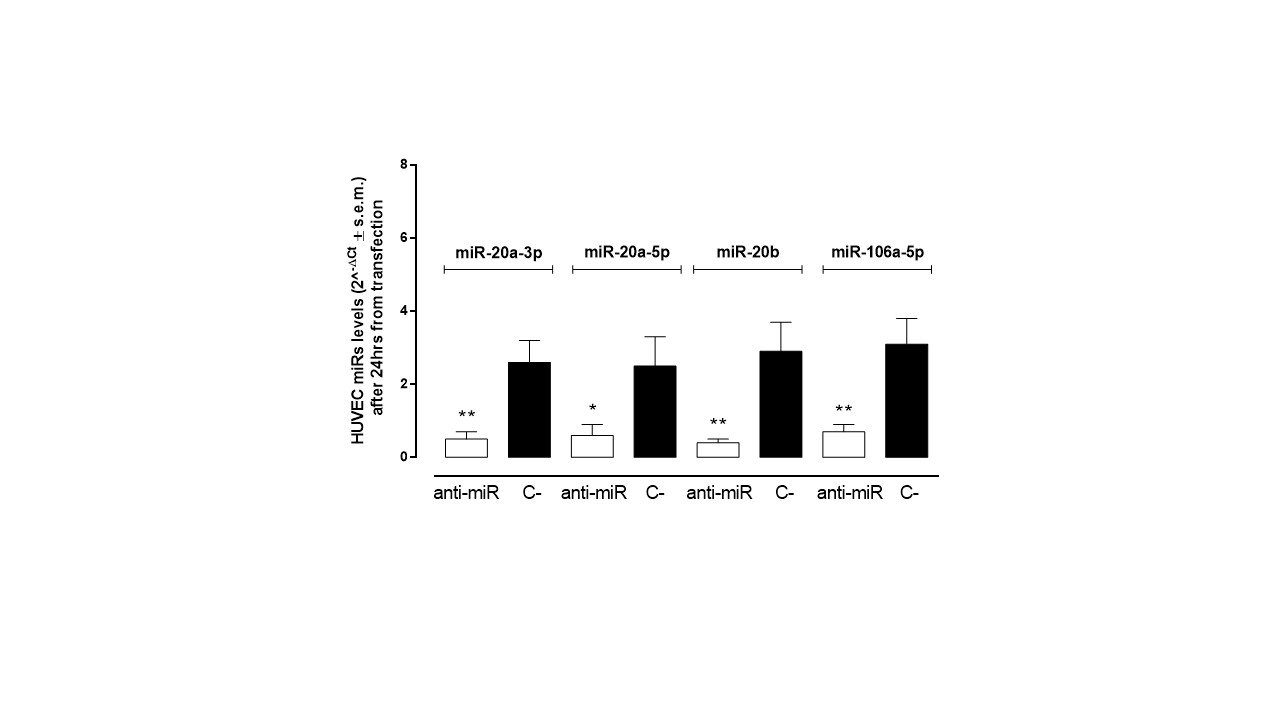
**

**Figure S5.** qRT-PCR for miR-20a-3p, miR-20a-5p, miR-20b, miR-106a-5p expression determination in HUVEC cells after a 24 hour-transfection with anti-hsa-miR-20a-5p, anti-hsa-miR-20a-3p, anti-hsa-miR-20b, anti-hsa-miR-106a-5p or negative control. Values are expressed as mean ± s.e.m. of n = 9 values, obtained from the triplicates of three independent experiments They were analyzed by one-way ANOVA followed by Bonferroni test’s.

**Figure S6
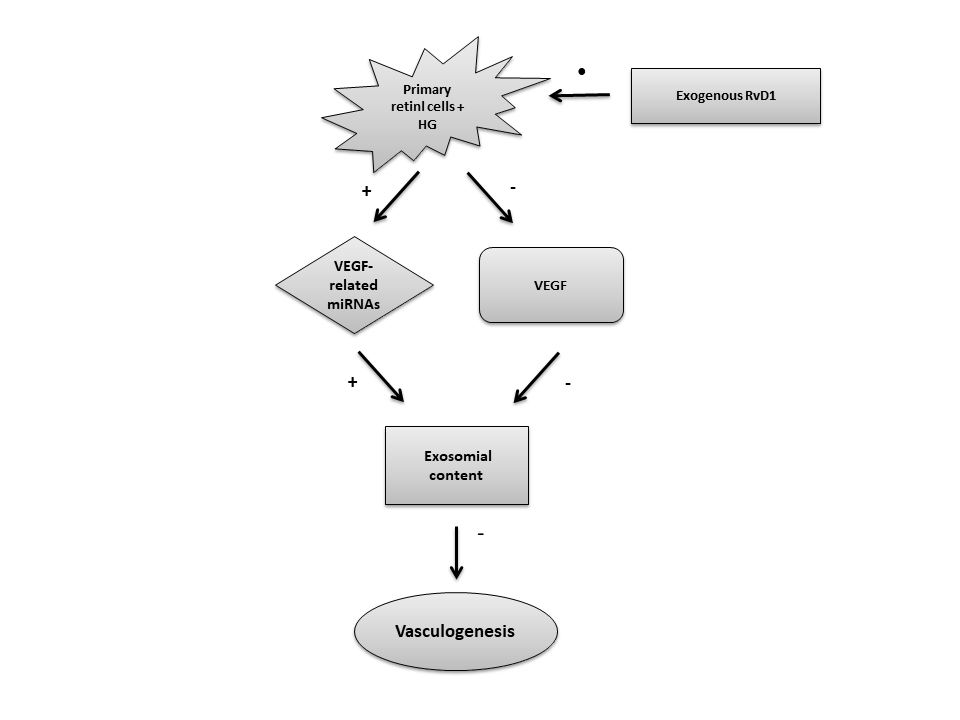
**

**Figure S6.** Graphical abstract. Diagram illustrating the effect of RvD1 against VEGF-related miRNAs of primary retinal cells challenged with high glucose. RvD1 is depicted as an anti-angiogenic compound, capable of decrease VEGF through the exosome-containing miRNAs and VEGF innovative pathway. It increases levels of anti-angiogenic miRNAs in primary retinal cells and in their exosomes. Accordingly, RvD1 decreases intracellular and exosomial VEGF content and the overall pro-angiogenic potential. HG = high glucose; RvD1= Resolvin D1; VEGF = vascular endothelial growth factor. ● = stimulation; + = increase; - = decrease.
